# Fundamental dissipation due to bound fermions in the zero-temperature limit

**DOI:** 10.1038/s41467-020-18499-1

**Published:** 2020-09-21

**Authors:** S. Autti, S. L. Ahlstrom, R. P. Haley, A. Jennings, G. R. Pickett, M. Poole, R. Schanen, A. A. Soldatov, V. Tsepelin, J. Vonka, T. Wilcox, A. J. Woods, D. E. Zmeev

**Affiliations:** 1 Department of Physics, Lancaster University, Lancaster, LA1 4YB UK; 2 P.L. Kapitza Institute for Physical Problems of RAS, Moscow, 119334 Russia; 3 Present Address: Paul Scherrer Institute, Forschungsstrasse 111, 5232 Villigen PSI, Switzerland; 4 Present Address: Department of Physics and NHMFL High B/T Facility, University of Florida, Gainesville, FL 32611 USA

**Keywords:** Phase transitions and critical phenomena, Quantum fluids and solids, Surfaces, interfaces and thin films

## Abstract

The ground state of a fermionic condensate is well protected against perturbations in the presence of an isotropic gap. Regions of gap suppression, surfaces and vortex cores which host Andreev-bound states, seemingly lift that strict protection. Here we show that in superfluid ^3^He the role of bound states is more subtle: when a macroscopic object moves in the superfluid at velocities exceeding the Landau critical velocity, little to no bulk pair breaking takes place, while the damping observed originates from the bound states covering the moving object. We identify two separate timescales that govern the bound state dynamics, one of them much longer than theoretically anticipated, and show that the bound states do not interact with bulk excitations.

## Abstract (web summary)

Understanding dynamics of fermionic bound states is important for their potential application in quantum devices. Here the authors study zero temperature dynamics and dissipation of fermions bound on a moving goal-post shaped wire in superfluid ^3^He-B.

## Introduction

Interfaces in fermionic condensates and the bound states hosted by them have recently attracted research focus in numerous condensed matter systems. Contemporary superfluid research is moving towards nanoscale probes^1,2^, nanoconfinement^3–5^ and microscopic dissipation mechanisms related to quantum turbulence^6,7^. Unconventional superconductivity in various materials^8^ and the dynamics of certain atomic BECs^9^ is characterised by the physics of bound excitations. Nanofabrication of superconducting hybrid structures has enabled tailoring bound states with desired properties. For instance, engineered superconducting systems containing Andreev-bound states (ABS) are used in studies of Majorana fermions^10,11^ aimed at producing components of, say, a quantum computer^12^. Understanding the dynamics of bound states is of central importance for these research directions.

A recent experiment showed, contrary to the classic textbook picture, that a macroscopic object moving quasi-uniformly in superfluid ^3^He-B can exceed the predicted Landau velocity ^13^ in the zero-temperature limit without destroying the condensate^14^. Phenomenologically, the dissipation experienced by objects moving rapidly can be explained in terms of the Lambert picture^15^. This scenario is based on assuming that there is a well-defined two-dimensional (2D) gapless spectrum of bound states available within roughly a coherence length of all surfaces, and that the fermions occupying these states can escape to the bulk promoted by macroscopic flow fields, thereby providing dissipation. This is clearly a simplification, but the model captures the main features of the experiment well enough for the presentation of the results. Simultaneously, the surface layer shields the bulk condensate from interacting with the moving object, preventing pair breaking even if the object’s velocity exceeds the Landau velocity. Therefore the bound states covering the surface of a fermionic condensate not only determine the properties of its surfaces, but ABS dynamics are also central in understanding one of the most elementary of bulk phenomena: the drag an object experiences when moving at constant velocity through the fermionic superfluid.

The existence of a gapless spectrum covering all surfaces in superfluid ^3^He is well-motivated by theoretical studies^16–19^, and supported by independent experimental evidence^18,20,21^. In terms of dynamics, however, the above picture still lacks rigorous theoretical justification. In particular, its critics have pointed out that thermalisation of the surface-bound states should be so fast, of the order of nanoseconds, that the equilibrium cannot be disturbed by the relatively slow motion (typically milliseconds) of a macroscopic moving object^22^. In this article, we provide experimental evidence to end the controversy. We show that such criticism correctly identifies only one of the two timescales that govern the dynamics of surface-bound states, and that this process is very fast as theoretically anticipated. Crucially, we also observe a second, much slower process, which allows for the surface states’ contribution to dissipation. Our experiment demonstrates, remarkably, that the observed dissipation is independent of the density of thermal bulk excitations, which justifies the assumption that surface-bound states are involved in the process in the first place.

## Results

An ideal experiment aimed at probing ABS-originating dissipation in superfluid ^3^He-B would be carried out at a temperature so low that thermal bulk excitations vanish completely. Otherwise, trivial collisions with them provide a thermal dissipation background. In our experiments, the sample temperature was between 140 μK and 240 μK. The superfluid transition temperature is *T*_c_ = 929 μK. Therefore the density of thermal bulk quasiparticles is not negligible, but it is low enough that they propagate ballistically, and their contribution to observed dissipation can be confidently subtracted.

In these conditions at saturated vapour pressure, the Landau critical velocity is *v*_L_ = Δ/*p*_F_ = 27 mm s^−1^ (Δ is the superfluid gap and *p*_F_ the Fermi momentum). We can reach and exceed this velocity in both oscillatory and constant-speed measurements by driving alternating (AC) or direct (DC) current through a goalpost-shaped superconducting wire (legs 25 mm, crossbar 9 mm, thickness 135 μm). An external magnetic field is oriented parallel to the legs of the wire, and the resulting Lorentz force on the crossbar moves the wire. In an AC measurement, the wire is moved by a sinusoidal current at the mechanical resonance frequency of the wire. This is how mechanical probes are traditionally operated in order to probe the dissipation in a liquid^23–25^. In a DC measurement, the drive current changes at a constant rate, resulting in a sweep of the probe wire from one side to the other at a (quasi) constant speed^14^. The wire is surrounded by a bolometric volume of superfluid ^3^He-B, and the drag force experienced by the wire is inferred from the number of emitted quasiparticles, seen as a heat pulse measured by the thermometers.

**Fig. 1:**
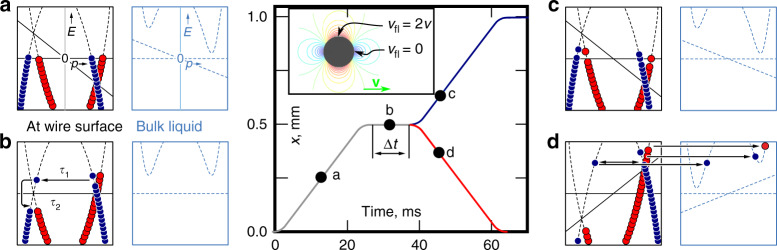
Schematic illustration of the measurement at zero temperature. (Centre) The crossbar of the wire is moved in two phases along direction labelled *x*. All ramps begin by accelerating the wire from zero to a constant velocity (here *v* = 40 mm s^−1^, gray line) and then back to zero. The magnitude of the resulting flow field, *v*_fl_, in the coordinate system of the wire is shown with contour lines in the inset. The ramp is then either repeated in the same direction (up ramp, blue line) or in the opposite direction (down ramp, red line). Bound-state dynamics are probed by varying the waiting time Δ*t* between the two phases. **a** The flow shifts both the bound-state energy spectrum, *E*(*p*), and the available bulk states as explained in the main text. After the acceleration, the population of bound quasiparticles (red circles) and quasiholes (blue circles) on the wire surface reaches a steady-state (point **a** along the wire trajectory). The schematic dispersion curves (black dash line at the surface, blue dash line in bulk) are drawn for the top (or bottom) generatrices of the wire, where the flow velocity is maximal. **b** During Δ*t*, momentum exchange with the wire surface allows the exchange of bound quasiparticle populations between the branches (*τ*_1_). Within a branch, the population relaxes with *τ*_2_. **c** During an up ramp, if Δ*t* ≲ *τ*_1_, the population imbalance results in less dissipation from quasiparticles escaping to bulk as compared with the first phase of motion. **d** In a down ramp, if Δ*t* ≲ *τ*_1_, the dissipation will be enhanced. Note that the bound-state dispersion depends on surface specularity, and the Dirac dispersion illustrated here is chosen for simplicity.

Let us study the consequences of a gapless spectrum of bound states covering the surface of the moving wire crossbar at a temperature so low that there are no thermal bulk excitations. The spectrum has an approximately direction-independent density of states and a relatively flat dispersion relation assuming the surface scatters quasiparticles diffusively, and a more Dirac-like dispersion relation with partial specularity^18^. For the purposes of the experiments presented here, the details of the dispersion relation are irrelevant. By default, the wire surface is diffusive, and tuning the scattering conditions using ^4^He preplating is discussed later. For the sake of simplicity, we have illustrated the measurement with Dirac dispersion curves in Fig. 1.

In the presence of macroscopic flow, both bulk and gapless surface dispersion curves for quasiparticles (and holes) shift according to  ±*v*_fl_*p*_F_ (in one dimension). Here, *v*_fl_ is the local flow velocity of the superfluid. In the frame of the crossbar, the bulk far away from the wire flows at *v*_fl_ = −*v*, where *v* is the velocity of the crossbar in the laboratory frame. Near the cylindrical crossbar, the flow is enhanced and reaches a maximum of *v*_fl_ = −2 *v*, assuming perfect potential flow. These two contributions narrow down the energy mismatch Δ between available bulk states and populated ABS states by 3 *vp*_F_, and the flow, therefore, enables ABS escape from the surface of the wire to the bulk when the wire moves faster than *v*_c_ = 1/3 ⋅ Δ/*p*_F_ = 9 mm s^−1^, the well-known critical velocity in AC measurements^26^. Strikingly, if *v* is held constant, the ABS spectrum finds equilibrium and the drag force disappears^14^.

The above-described difference between oscillatory and uniform motion must arise from the time constants that govern the ABS escape process. Basically, the repeating reversal of direction in oscillatory motion replenishes the reserve of quasiparticles that can escape to bulk when the velocity exceeds the critical velocity, while in uniform motion such replenishment is not available. If we compare equally long and otherwise identical trajectories where velocity is either reversed or not, the reversal should increase the observed dissipation. In the absence of the bound-state contribution, such directional dependence is not expected^14^.

In order to experimentally substantiate this picture, and to remove the effect of the finite density of thermal bulk excitations that cannot be avoided in experiments, we move the wire crossbar in two phases (Fig. 1). Each measurement starts from standstill long enough to remove any history dependence. The wire is then quickly accelerated to *v*, the velocity is kept constant for 0.5 mm distance, before reducing the velocity back to zero^27^. The acceleration time was varied from 3 ms to 5 ms, and the obtained results were independent of it. After time Δ*t*, the ramp is repeated from this new starting location with either *v* (“up ramp”) or  −*v* (“down ramp”). This allows subtracting the dissipation measured for up ramps *Q*_up_ from that measured for down ramps *Q*_down_, Δ*Q* = *Q*_down_ − *Q*_up_. As described in detail in “Methods”, the dissipation due to ambient bulk quasiparticles in Δ*Q* is cancelled assuming the up and down trajectories are equally long.

The above measurement shows that bound-state dissipation is characterised by two regimes: First, the difference in the measured dissipation, Δ*Q*, is zero when *v* ≤ 9 mm s^−1^ (see “Methods”). This implies that, in the zero-temperature limit, dissipation vanishes below the critical velocity *v*_c_ = 9 mm s^−1^. Second, for velocities higher than 9 mm s^−1^ at Δ*t* = 0, up ramps experience less dissipation than down ramps. Phenomenologically we find that $\Delta Q\propto {((v-{v}_{{\rm{c}}})/{v}_{{\rm{c}}})}^{2.5}$. We can only measure this velocity dependence in detail when the sample is preplated with ^4^He. This could in principle limit applying the obtained results on the quasiparticle dynamics where no ^4^He is present. However, we emphasise that *v*_c_ = 9 mm s^−1^ is the AC critical velocity, observed here both with and without ^4^He, confirming that the results of the DC measurement directly apply to dissipation experienced by moving objects in superfluid ^3^He-B in general. This shows that direct bulk pair breaking is replaced by a much weaker drag force originating from surface-bound quasiparticles, as speculated earlier^14^. The velocity dependence is discussed in more detail in “Methods”.

**Fig. 2:**
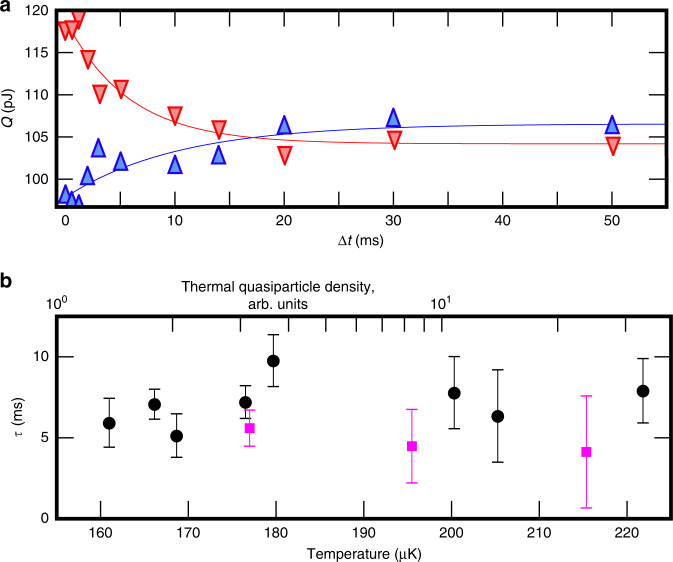
Bound-state dynamics. **a** Measured dissipation as a function of Δ*t* for up ramps (blue upward triangles) and down ramps (red downward triangles) reveals the characteristic time of bound-state dynamics on the wire surface. The solid lines are exponential fits to the data. At large waiting times Δ*Q* ≈ − 2.6 pJ, as expected due to the hysteretic contribution analysed in Fig. 4. **b** The fitted time constants with ^4^He preplating (magenta squares) and without it (black circles) are independent of temperature, *τ* = 6 ± 3 ms. The relative change in thermal quasiparticle density is shown on the top axis. All data in this figure were measured at *H* = 130 mT and *v* = 45 mm s^−1^. Error bars indicate the 68% confidence interval for the exponential fits.

We can now vary Δ*t* to measure the dynamics of bound states on the wire surface (Fig. 2). The difference in dissipation, Δ*Q*, disappears exponentially as $\exp (-\Delta t/\tau )$, where *τ* ≈ 6 ms in the measured temperature range of 160–230 μK. Within this range of temperatures, the thermal bulk quasiparticle density varies by almost two orders of magnitude, while measured *τ* is constant. Together with the above observations, this justifies the assumption that bound quasiparticles are responsible for the dissipation. It also rules out any direct interaction between surface-bound and bulk quasiparticles as the source of thermalisation.

The dynamics of bound quasiparticles on the wire surface can be described by two time constants in our toy model. The first one, *τ*_1_, gives the probability of the inter-branch process where a quasiparticle scatters from the wire exchanging momentum going from, say, *p* to  −*p*. This process enables drag, as the exit channel to bulk is open only in the direction of wire motion while only quasiparticles with the momentum in the opposite direction gain energy due to the flow. That is, drag is produced by those quasiparticles that escape to bulk right after scattering with the wire, removing momentum from the wire. The second time constant, *τ*_2_, describes the rate at which quasiparticles that did not escape to bulk relax within each dispersion branch to the thermal equilibrium distribution distorted by process one. If *τ*_2_ = *∞*, then the imbalance produced by moving the wire and stopping in the middle, measured by comparing up and down ramps, disappears diffusively as determined by *τ*_1_. Assuming *τ*_2_ = 0, the imbalance still disappears at the rate given by *τ*_1_, but this time the process is deterministic. Therefore, regardless of *τ*_2_, we have measured *τ*_1_ ≈ *τ* = 6 ms.

**Fig. 3:**
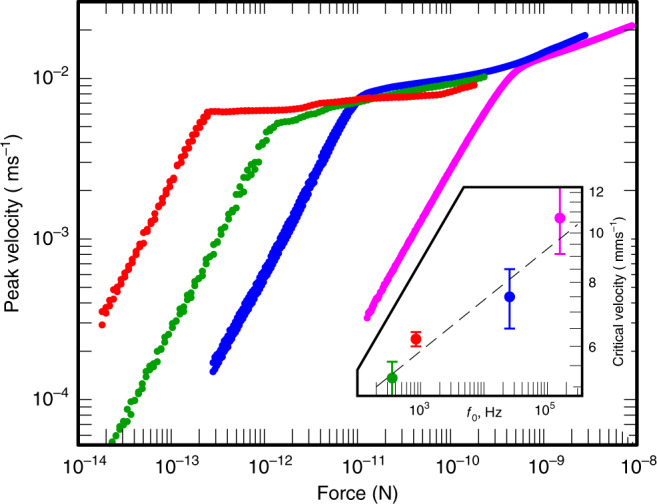
AC critical velocity measurements as a function of frequency. In AC measurements, critical velocity *v*_c_ is seen as a sharp increase in the force needed to increase peak oscillation velocity above the critical velocity. The exact critical velocity depends on details of the flow field around the probe, and therefore on the shape of the object. The measured probes are in order from left to right: a 1-μm thick vibrating wire operating at 843 Hz (red points, measured at 110 μK), a 4.5-μm thick vibrating wire at 355 Hz (green points, 110 μK), a custom-made quartz tuning fork at 25.7 kHz with prong length 1.75 mm and prong cross-section 50 × 90 μm (blue points, 120 μK), and an overtone of the same fork at 158 kHz (magenta points, 110 μK). The inset shows critical velocities extracted from the main figure. Error bars correspond to the uncertainty in locating the critical velocity in the main figure. Black dash line is a guide to the eye that corresponds to ${v}_{{\rm{c}}}\propto {f}_{0}^{0.1}$, illustrating the increase of *v*_c_ with frequency.

It is also possible to set an experimental upper bound for *τ*_2_. We have measured the AC critical velocity of a set of probes with resonance frequencies *f*_0_ ranging from 350 Hz to 158 kHz (Fig. 3). The critical velocity is seen as kink in the velocity of the probe measured as a function of force. It corresponds to a sudden increase in the force required to increase the velocity by a given amount. As speculated by Lambert^15^, when *f*_0_ > 1/*τ*_2_, one should see a clear reduction in the observed AC critical velocity as quasiparticles would be able to “climb up” the dispersion curves diffusively by scattering back and forth between *p* and  −*p*. For instance, by a two-step process, one would get escape to bulk starting at 4.5 mm s^−1^, and so on.

We observe no critical velocity reduction up to *f*_0_ = 158 kHz. In fact, the observed critical velocity slowly and smoothly increases as a function of the resonance frequency. This happens despite the varying geometries of the four probes used: the two low-frequency probes are vibrating superconducting wires, while the two kHz probes are the first harmonic and an overtone of a custom-made quartz tuning fork with smooth surfaces. While studying the reason for the observed slow increase of *v*_c_ systematically remains a task for the future, this result implies that *τ*_2_ ≲ 1/*f*_0_ ≈10 μs. We believe that *τ*_2_ describes the fast thermalisation process anticipated in ref. ^22^.

Surface specularity may play an important role in the bound-state escape process. In particular, the critical velocity *v*_c_ predicted by the phenomenological toy model depends on the bound-state gap for quasiparticles with in-plane momentum, Δ_∥_, which is zero for a diffusively scattering surface and obtains the bulk value when the surface becomes fully specular^18^. In the one-dimensional picture, the flow couples to bound quasiparticles with momentum in the plane of the surface. The predicted *v*_c_ therefore eventually approaches *v*_L_ = 27 mm s^−1^ with increasing specularity. Also, one would expect that *τ*_1_ depends on specularity as it describes the process of quasiparticle scattering with the wire.

The surface scattering can be tuned from diffuse to specular continuously by preplating the sample with slightly over two monolayers of ^4^He^28^. It is possible that ^4^He coverage also changes the scattering qualitatively by removing magnetic spin exchange with the surface layer^29^. However, reaching fully specular scattering requires a two-dimensional superfluid layer of ^4^He, which flows to the coldest part of the sample container (the heat sink) and cannot therefore be stabilised on the wire in our experiment^5^. We have resorted to measuring the effect of adding ~2.5 monolayers of solid ^4^He on the wire surface (and all other surfaces in the sample container). The amount of ^4^He to be added was estimated based on the dimensions of the sample container, the known surface area of the heat exchanger sinters^30^, and the known density of ^4^He monolayers^31^. This estimate was then favourably compared with the amount of ^4^He needed to be added to the originally-empty sample container at 4 K temperature to create a small equilibrium pressure, corresponding to saturated solidification on the surfaces.

While adding ^4^He causes the heat exchangers to become less efficient by a factor of two^32^, we observe very little change in the bound-state dynamics or dissipation. First, *τ*_1_ remains  ≈5 ms within the scatter of the data. This shows that the quasiparticle escape process is robust against at least small changes in the scattering conditions. Second, the measured AC critical velocity is 9 mm s^−1^ with and without ^4^He. This implies that the bound-state dispersion does not change dramatically. Based on Fig. 4 in ref. ^18^, this suggests that the surface specularity *S* obtained was smaller than *S* = 0.8, and probably between *S* = 0.2 and *S* = 0.5. We therefore conclude that the observed DC velocity dependence is (qualitatively) universal at least up to the obtained specularity. Measuring the precise amount of ^4^He on the surfaces is possible indirectly using nuclear magnetic resonance techniques as ^4^He replaces the paramagnetic layers of solid ^3^He otherwise covering all surfaces^5,33–35^, but building a new experiment with the necessary instruments for that remains a task for the future.

**Fig. 4:**
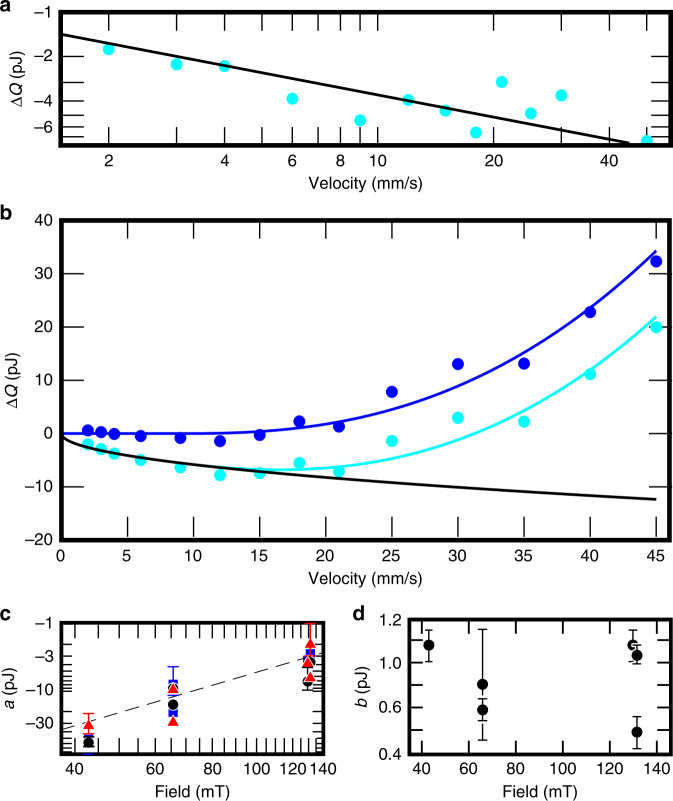
Bound-state dissipation and critical velocity. **a** The measured hysteretic difference in dissipation with Δ*t* = 50 ms (cyan circles) follows the empirical power law $\Delta Q=a{(v/{v}_{{\rm{c}}})}^{1/2}$ (solid line), as clearly seen in log scale. **b** When Δ*t* = 0 (cyan circles), the hysteretic contribution can be removed by fitting the data at *v* ≤ 9 mm s^−1^ with the above power law (solid black line). After subtracting this fit, the data (blue circles) follows $\Delta Q=b{((v-{v}_{{\rm{c}}})/{v}_{{\rm{c}}})}^{2.5}$ (solid blue line) above *v*_c_ = 9 mm s^−1^, and Δ*Q* = 0 for *v* <  *v*_c_, as explained in the text. The sum of the two fits is shown by the cyan line. The fitted values of *a* are shown in panel (**c**): Fits to data where Δ*t* = 0 are shown with black circles, Δ*t* = 50 ms corresponds to blue squares, and Δ*t* = 100 ms is shown as red triangles. The dashed line is a guide to the eye that corresponds to *a* = −5.0/*H*^2^nJ mT^2^. Panel (**d**) shows fitted values of *b* (black circles), which within the scatter of the data are independent of *H* as expected. The temperature varied from 150 μK to 190 μK in these measurements. All data in this figure were obtained with ^4^He preplating. Selected error bars in panel **c** correspond to one standard deviation of the fits to illustrate the point-wise scatter (omitted error estimates are similar and can be found in the data container, see the data availability statement), and in panel **d** to the 68% confidence interval of the fits.

## Discussion

In conclusion, our experiments confirm that quasiparticles (and holes) bound to the region of gap suppression near solid surfaces in the B phase of superfluid ^3^He are responsible for the zero-temperature dissipation experienced by macroscopic objects moving in the superfluid. The dissipation begins when the flow velocity is sufficient for releasing the bound quasiparticles to the bulk of the superfluid. We have demonstrated experimentally that the bound states re-equilibrate through a two-stage process involving a fast thermalisation step with *τ*_2_ < 10 μs, but importantly also a theoretically unforeseen slow scattering step characterised by *τ*_1_ = 6 ms. To be clear, *τ*_1_ is the effective time constant describing the process where the quasiparticle momentum is changed from **p**_F_ to −**p**_F_. In a proper two-dimensional treatment of the process that *τ*_1_ emerges from, this may involve a large number of scattering events which collectively allow the quasiparticle to eventually find the very opposite momentum. We speculate that this process is potentially the underlying reason as to why *τ*_1_ is not of the order of nanoseconds. Furthermore, we vary the bulk quasiparticle concentration to show that the bulk states do not contribute to bound-state dynamics on any observable timescale. Therefore our results can be readily generalised to the description of transient phenomena in 2-dimensional Dirac systems such as graphene^36^, if the escape process to bulk is neglected. It is worth emphasising that the bulk escape process is temperature independent at low temperatures and, hence, provides dissipation even in the zero-temperature limit.

We acknowledge the preliminary nature of the bound-state model presented above. In particular, it remains an open question how exactly bound-state dynamics should be described in a full two-dimensional model of the wire surface with a flow velocity distribution, a theoretically justifiable bound state spectrum (not fully gapless), diffusion or transport between various parts of the surface etc. Competing theoretical suggestions are sparse, but there have been some discussions related to a layer of vortices covering the wire acting as a buffer and therefore masking the Landau velocity^22^. On the other hand, theoretical work on superflows exceeding the Landau velocity has recently been published studying other systems, such as polaron–polaritons^37^ and graphene^38^. For these reasons, it remains of interest to provide additional experimental insight. For example, one could change the Landau velocity by studying flow in a confined geometry, say, in the recently-discovered polar phase of superfluid ^3^He^3^. The gap spectrum of the polar phase contains a nodal line, meaning that the Landau speed limit is zero in that plane. On the other hand, it seems essential to enhance the experiment presented in this article by building a detector or spectrometer fast enough to distinguish the dissipation associated with the separate phases of the wire motion. This requires designing and devising nano-sized instruments^1,2^.

## Methods

An ideal experiment aimed at probing bound-state-originating dissipation in superfluid ^3^He-B would be carried out at a temperature so low that thermal bulk excitations are rare enough to be neglected completely. Otherwise, trivial collisions with them provide a thermal dissipation background. In our experiments, we used a nested nuclear demagnetisation cryostat^39^, capable of holding the sample temperature anywhere between 140 μK and 240 μK for several days. The temperature was measured with a quartz tuning fork^23^, and a vibrating wire thermometer^24,25^. Pressure in all measurements was the saturated vapour pressure, corresponding to superfluid transition temperature *T*_c_ = 929 μK. Therefore the density of thermal bulk quasiparticles is not negligible, but it is low enough so that they propagate ballistically, and their contribution to the observed dissipation can be subtracted. We can vary the thermal quasiparticle density by two orders of magnitude within the ballistic regime. Subtracting the thermal quasiparticle contribution is robust and reliable because the gap spectrum of the B phase is isotropic in zero magnetic field, and only slightly distorted in the small external magnetic field.

The main probe used in the experiment is the goalpost-shaped wire. The bolometric volume that surrounds the wire is calibrated by resonant AC measurements^40^ by fitting the measured calibration data to known BCS heat capacity using the effective volume of the sample as a fitting parameter. The fitted volume is 16 cm^3^, which falls between the free volume of the sample container, 15 cm^3^ and the total volume of the sample container, including the volume within the heat exchangers, 32 cm^3^.

We simultaneously monitor the position of the wire by picking up a high-frequency signal mixed in with the driving current using nearby coils. This method is not sensitive enough for measuring the drag force^41^, but together with the known wall-to-wall distance (≈10 mm) it calibrates the range of motion. Finally, we record the induced voltage across the wire with a four-point measurement. The induced voltage reveals whether the main AC resonance or some higher mode of oscillation is excited by the DC drive, allowing us to ensure that dissipation due to these modes was minimised during all measurements.

We access zero-temperature dissipation by moving the wire crossbar in two phases (Fig. 1). Each measurement starts from standstill long enough to remove any history dependence. The wire is then quickly accelerated to *v*, the velocity is kept constant for 0.5 mm distance, before decelerating back to zero. After time Δ*t*, the ramp is repeated from this new starting location with either *v* (up ramp) or  −*v* (down ramp). This allows subtracting the dissipation measured for up ramps *Q*_up_ from that measured for down ramps *Q*_down_, Δ*Q* = *Q*_down_ − *Q*_up_.

Ideally, both the trajectories would meet identical dissipation from the scattering of thermal quasiparticles in bulk. Subtracting the dissipation measured for up ramps *Q*_up_ from that measured for down ramps *Q*_down_ would leave only the ABS contribution Δ*Q* = *Q*_down_ −  *Q*_up_, which for long enough Δ*t* is expected to be zero. In practice, the motion of the goalpost-shaped superconducting wire is hysteretic^27^ due to the motion of flux lines or some other dissipation in the superconducting wire. Flux line motion, for example, would be driven by variation of the current used for moving the wire in the magnetic field, or by the effective change of direction of the external magnetic field when the legs of the wire are bent^42–44^.

This dissipation is not seen in the temperature of the surrounding bolometer because the coupling of the phonons in the metallic wire to the superfluid is very weak due to the Kapitza resistance. However, the hysteretic motion has an indirect consequence for our measurements: the crossbar travels a shorter distance with down ramps than with up ramps, and as a result, scatters a different amount of ambient quasiparticles. Therefore, in the absence of other contributions, Δ*Q* < 0. Phenomenologically, the hysteretic contribution follows $\Delta Q=a{(v/{v}_{{\rm{c}}})}^{1/2}$ (Fig. 4a, b), where *a* ∝ (Δ*I*)^2^ (Fig. 4c). That is, the force moving the wire is kept constant in all the measurements (for a given velocity *v*), and measurements were carried out in varying magnetic fields *H*. Fixing the force means that the change in the current passed through the wire *ΔI* ∝ 1/*H*, and therefore *a* ∝ (*H*)^−2^. A detailed study of the origin of this effect is beyond the scope of the present work.

Once the hysteretic contribution is subtracted, Δ*Q* = 0 when *v* ≤ 9 mm s^−1^ (Fig. 4b), corresponding to zero dissipation from the bound states. For velocities higher than 9 mm s^−1^ at Δ*t* = 0, up ramps experience less dissipation than down ramps. In this region, we find that $\Delta Q=b{((v-{v}_{{\rm{c}}})/{v}_{{\rm{c}}})}^{p}$ with *p* = 2.5 ± 0.5 and *b* ≈ 1.0 pJ. The data in Fig. 4d are fitted with fixed *p* = 2.5 in order to show that *b* has no magnetic field dependence. That is, all the field dependence is contained in parameter *a*, as expected. Lambert^15^ predicts that, in a planar geometry, the instantaneous flux of energy follows *p* = 5 when (*v* − *v*_c_)/*v*_c_ is small, and *p* = 2 when (*v* − *v*_c_)/*v*_c_ is large. Our measured value falls in the predicted range, but this calculation neglects the possibility that the bound quasiparticle reserve is completely exhausted, and is therefore not directly applicable to the DC measurements presented here.

Note that the measurement shown in Fig. 4 was obtained with ^4^He preplating, which changes the surface specularity and therefore, the bound-state spectrum. While this does not increase the obtained critical velocity, it is possible that *p* is different with no ^4^He. Detailed analysis of the velocity-dependence data obtained in DC measurements without ^4^He preplating is hindered by the coincidence that without ^4^He the signal becomes too small to resolve at low velocities. That is, ^4^He preplating makes the heat exchangers less efficient, which increases the signal. This allows us to access also velocities below *v*_c_, which is crucial for the above analysis owing to the hysteresis effect.

## Data Availability

All the data in this paper are available at 10.17635/lancaster/researchdata/356, including descriptions of the data sets.
